# Supplementary figures and images for: Spatiotemporal patterns of tuberculosis in urban slums and urban–rural transition zones: evidence from Tétouan, Morocco, 2019–2023
Source: PLOS Glob Public Health. 2026 Apr 20;6(4):e0006315. doi: 10.1371/journal.pgph.0006315 (PMC13095008; doi:10.1371/journal.pgph.0006315)

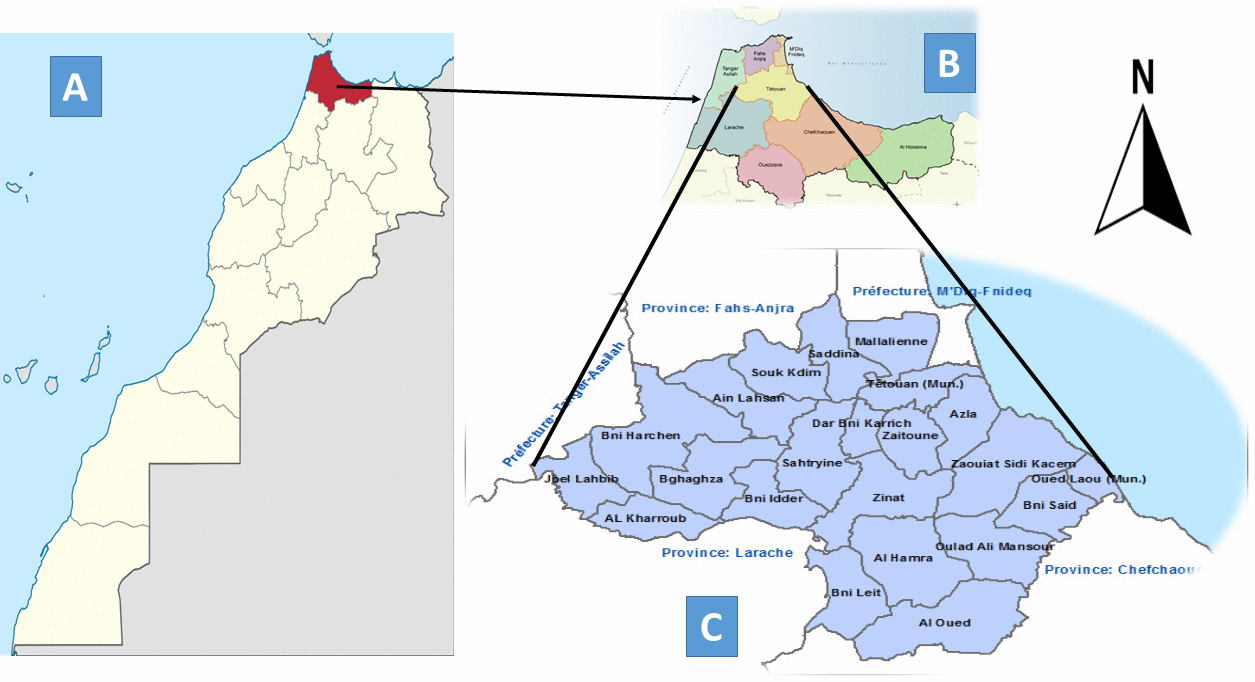

Supplement: S1 Fig — A: Map of Morocco with regional organization; B: Region of Tangier-Tétouan-Al Hoceima; C: Province of Tétouan divided into communes. (TIF) [file pgph.0006315.s001.tif]
