# Supplementary material for: Spatiotemporal patterns of tuberculosis in urban slums and urban–rural transition zones: evidence from Tétouan, Morocco, 2019–2023
Source: PLOS Glob Public Health. 2026 Apr 20;6(4):e0006315. doi: 10.1371/journal.pgph.0006315 (PMC13095008; doi:10.1371/journal.pgph.0006315)
Supplement: S1 Table — This table summarizes the administrative organization of Tétouan Province. Urban areas include Tétouan and Oued Laou municipalities. Tétouan is subdivided into the Old Medina (historic, high-density neighborhoods including slums) and the New Medina (modernized areas with improved housing). Oued Laou, though urban, has a small population (11,690 in 2014), a limited area (~32.8 km2), no district subdivision, and one primary healthcare center. Rural areas consist of 20 surrounding communes. (DOCX) [file pgph.0006315.s002.docx]

| S1 Table: Administrative Division of Tétouan Province, by the High Commission for Planning, Tangier-Tétouan-Al Hoceima Regional Directorate, 2018 | | | |
| --- | --- | --- | --- |
| Area | **Commune/ Municipality (Mu.)** | **District** | |
| Urban  (two Municipalities ) | **Mu. of Tetouan*** | **Old medina**  **(Slums)** | Bab Tout, Mellah,  Dersa III, Sidi Frij,  Samsa, El Kassaba, Dersa I. |
|  |  | **New medina**  **(Modern area)** | M'hannech, Boujarrah, C.Scolaire.  My Hassan, Tabola, Nakata, Sidi Talha, Coelma, Dar Murcia, Korrat Sbaa. Touilaa. |
|  | **Mu. of Oued Laou**** |  | |
| Rural  (20 commune) | Ben Karrich, Zinat, Ben Idder, Sahtriyine, Bghaghza, Alhamra, Bni Lait, Oulad Ali Mansour, El Oued, Malaliyene, Saddina, Sebt Kdim/souk kdim, Fondaq/Ain Lahsan, Bni Harchane, Jbel Hbib, El Kharoub, Beni Said, Z.S.Kacem, Zaitoune, Azla. | | |
| * Tétouan retains its ancient Medina, which is entirely enclosed by the historic city wall. All districts of the Old Medina are located within this wall, representing the high-density historic neighborhoods, including informal and slum-like areas. Districts located outside the wall constitute the New (or Modern) Medina, which developed later and is characterized by more recent urban planning and comparatively improved housing and hygienic conditions.  ** Oued Laou is administratively classified as an urban municipality but has a small population (11,690 inhabitants according to the 2014 national census), limited area (≈32.8 km²), no district subdivision, and a single primary healthcare center, and it is located 44.2 km from Tétouan. | | | |
