# Supplementary material for: Spatiotemporal patterns of tuberculosis in urban slums and urban–rural transition zones: evidence from Tétouan, Morocco, 2019–2023
Source: PLOS Glob Public Health. 2026 Apr 20;6(4):e0006315. doi: 10.1371/journal.pgph.0006315 (PMC13095008; doi:10.1371/journal.pgph.0006315)
Supplement: S2 Table — The table presents annual crude tuberculosis incidence rates in Tétouan Province. Urban areas include Tétouan and Oued Laou municipalities. Tétouan municipality is divided into the Old Medina (historic, high-density neighborhoods, all located within the ancient walled Medina) and the New Medina (modern areas with more recent urban planning). Oued Laou, though urban, has a small population, limited area (~32.8 km2), no district subdivision, and one primary healthcare center. Rural areas comprise 20 surrounding communes. Rates are expressed per 100,000 population; zero indicates no reported cases. The 2019–2023 values represent the average annual incidence over the five-year period, calculated using reported cases and population estimates from the Moroccan High Planning Commission (HCP) and regional health records. (DOCX) [file pgph.0006315.s003.docx]

| ****S2**** ****Table.**** Annual crude tuberculosis incidence rates (per 100,000 population) by communes and urban district in Tétouan Province, Morocco, 2019–2023 | | | | | | | | | |
| --- | --- | --- | --- | --- | --- | --- | --- | --- | --- |
| Area | **Commune/ Municipality** | **District** | | **Year** | | | | | |
|  |  |  |  | **2019** | **2020** | **2021** | **2022** | **2023** | **2019-2023*** |
| Urban | **Mu. of T**é**touan** | **Old medina**  **(Slums)** | **Bab Tout** | 321.95 | 306.22 | 354.68 | 258.60 | 255.99 | 298.99 |
|  |  |  | **Mellah** | 306.88 | 310.88 | 292.52 | 229.56 | 177.84 | 262.44 |
|  |  |  | **Dersa III** | 188.30 | 211.59 | 198.87 | 186.94 | 150.96 | 187.11 |
|  |  |  | **Sidi Frij** | 179.31 | 93.96 | 142.60 | 218.06 | 190.47 | 165.42 |
|  |  |  | **Samsa** | 146.49 | 155.99 | 165.74 | 172.07 | 157.02 | 159.72 |
|  |  |  | **El Kassaba** | 174.14 | 135.55 | 127.32 | 155.98 | 178.16 | 154.96 |
|  |  |  | **Dersa I** | 156.86 | 149.58 | 109.21 | 129.67 | 133.71 | 135.64 |
|  |  | **New medina**  **(Modern area)** | **Touilaa** | 248.91 | 194.81 | 187.34 | 112.20 | 144.87 | 179.10 |
|  |  |  | **M'hannech** | 161.57 | 134.20 | 162.64 | 165.17 | 150.93 | 154.93 |
|  |  |  | **Boujarrah** | 116.64 | 147.10 | 134.78 | 122.25 | 162.27 | 136.72 |
|  |  |  | **C.Scolaire** | 139.46 | 131.76 | 107.15 | 149.10 | 144.32 | 134.42 |
|  |  |  | **My Hassan** | 128.46 | 136.71 | 133.73 | 91.11 | 109.52 | 119.77 |
|  |  |  | **Tabola** | 143.82 | 94.10 | 105.74 | 117.18 | 124.28 | 116.79 |
|  |  |  | **Nakata** | 125.00 | 114.68 | 99.71 | 103.15 | 119.86 | 112.43 |
|  |  |  | **Sidi Talha** | 152.25 | 143.34 | 113.99 | 106.16 | 32.84 | 109.00 |
|  |  |  | **Coelma** | 132.28 | 56.34 | 74.50 | 110.58 | 133.79 | 101.64 |
|  |  |  | **Dar Murcia** | 109.59 | 101.79 | 52.65 | 85.24 | 103.14 | 90.00 |
|  |  |  | **Korrat Sbaa** | 131.15 | 57.87 | 57.32 | 70.90 | 129.83 | 89.46 |
|  |  | **All districts** | | 155.60 | 136.75 | 132.55 | 131.63 | 139.01 | 139.03 |
|  | **Mu. Oued Laou** |  | | 95.69 | 122.07 | 82.46 | 18.13 | 62.82 | 75.67 |
| Rural | **Beni Karrich** | |  | 101.24 | 200.89 | 149.18 | 63.60 | 116.09 | 125.69 |
|  | **Zinat** | |  | 25.74 | 89.27 | 24.20 | 24.07 | 59.91 | 44.41 |
|  | **Ben Idder** | |  | 21.44 | 63.37 | 19.38 | 0.00 | 0.00 | 20.04 |
|  | **Sahtriyine** | |  | 50.66 | 37.66 | 0.00 | 59.28 | 82.63 | 46.16 |
|  | **Bghaghza** | |  | 14.68 | 0.00 | 13.69 | 40.84 | 81.33 | 30.79 |
|  | **Alhamra** | |  | 91.25 | 90.68 | 69.85 | 60.79 | 69.18 | 76.08 |
|  | **Bni Lait** | |  | 99.08 | 0.00 | 54.14 | 53.85 | 17.87 | 44.66 |
|  | **Oulad Ali Mansour** | |  | 81.11 | 0.00 | 18.43 | 36.66 | 54.75 | 38.03 |
|  | **El Oued** | |  | 27.88 | 9.23 | 8.89 | 70.71 | 44.00 | 32.42 |
|  | **Malaliyene** | |  | 113.38 | 78.74 | 75.15 | 128.14 | 63.80 | 91.71 |
|  | **Saddina** | |  | 101.92 | 72.07 | 40.74 | 27.02 | 67.25 | 61.10 |
|  | **Sebt Kdim/ souk kdim** | |  | 52.71 | 104.45 | 86.59 | 49.22 | 98.01 | 78.24 |
|  | **Fondaq/ Ain Lahsan** | |  | 92.65 | 45.83 | 57.38 | 42.81 | 99.46 | 67.57 |
|  | **Bni Harchane** | |  | 15.72 | 0.00 | 14.58 | 29.01 | 57.78 | 23.90 |
|  | **Jbel Hbib** | |  | 22.34 | 43.99 | 20.11 | 0.00 | 79.68 | 33.31 |
|  | **El Kharoub** | |  | 45.13 | 87.49 | 36.89 | 36.69 | 73.07 | 55.22 |
|  | **Beni Said** | |  | 55.46 | 0.00 | 10.52 | 31.38 | 72.90 | 40.21 |
|  | **Z.S.Kacem** | |  | 52.68 | 17.45 | 16.83 | 41.85 | 66.67 | 39.20 |
|  | **Zaitoune** | |  | 148.55 | 104.22 | 117.27 | 183.30 | 215.69 | 154.36 |
|  | **Azla** | |  | 76.85 | 100.08 | 97.64 | 142.83 | 193.41 | 122.69 |
| * The 2019–2023 values correspond to the average annual incidence over the five-year study period. Tétouan municipality (urban area) is presented at the district level. Zero values indicate years with no reported cases. Incidence rates are expressed as crude annual rates per 100.000 population. Rates were calculated using all reported cases and population denominators from the Moroccan High Planning Commission (HCP) 2019 estimates and regional public health records. | | | | | | | | | |
