# Supplementary material for: Spatiotemporal patterns of tuberculosis in urban slums and urban–rural transition zones: evidence from Tétouan, Morocco, 2019–2023
Source: PLOS Glob Public Health. 2026 Apr 20;6(4):e0006315. doi: 10.1371/journal.pgph.0006315 (PMC13095008; doi:10.1371/journal.pgph.0006315)
Supplement: S1 Text — Legend: This file provides additional details on case definitions, inclusion criteria, handling of missing data, and analytical methods (time-series, spatial, and spatio-temporal analyses) used in the study. (DOCX) [file pgph.0006315.s007.docx]

**Extended materials, methods, and operational definitions for tuberculosis surveillance and spatial analyses in Tétouan Province, 2019–2023**

**Case definitions:**

Tuberculosis (TB) cases were classified according to Moroccan National Tuberculosis Control Program (PNLAT) and World Health Organization guidelines. **Pulmonary TB** (PTB) included patients with clinical and radiological evidence of TB involving the lungs. PTB was considered bacteriologically confirmed when GeneXpert Ultra MTB/RIF assay was positive (which has been implemented as the primary diagnostic test since 2017 in Tetouan). Smear microscopy and culture were used mainly for bacteriological follow-up during treatment. **Extrapulmonary TB** (EPTB) included organ-specific TB diagnosed based on clinical presentation, with confirmation by histopathology, imaging, or bacteriological testing when available. Cases with **concurrent pulmonary and extrapulmonary involvement** were classified as **PTB+EPTB (concurrent)**.

​Treatment history was classified as ***new*** if the patient had never been treated for TB or had received anti-TB treatment for less than one month. Patients were classified as ***previously treated (retreatment)*** if they had received one month or more of anti-TB treatment in the past and were notified again during the study period. Retreatment cases were further subclassified as: **relapse** (previously declared cured or treatment completed and subsequently diagnosed again with TB); **treatment after loss to follow-up** (previously treated and declared lost to follow-up at the end of the most recent treatment episode and later re-registered for treatment); or **treatment after failure** (re-registered for treatment after being declared treatment failure at the end of the most recent treatment episode).

​Treatment outcomes were recorded using standard programmatic definitions. **Cure** was defined as bacteriologically confirmed PTB at treatment initiation with documented bacteriological negativity in the last month of treatment. **Treatment completed** was defined as completion of treatment without the bacteriological results required to classify the case as cured. The overall **treatment success** rate was defined as the sum of cured and treatment-completed cases. **Treatment failure** was defined as sputum smear or culture positivity at month five or later during treatment. **Death** was defined as death from any cause related to tuberculosis during treatment. **Loss to follow-up** was defined as treatment interruption for two consecutive months or more. **Transferred out/not evaluated** was defined as transfer to another reporting unit with treatment outcome unknown to the reporting unit. **Diagnostic error** was defined as cases initially registered as TB but subsequently reclassified as not TB by the treating service.

**Inclusion and exclusion criteria:**

All tuberculosis cases notified to the Moroccan National Tuberculosis Control Program (PNLAT) registry in Tétouan Province between 2019 and 2023 were included in the study (N = 3,614). No notified cases were excluded due to missing information. Missing data were limited and variable-specific; therefore, each analysis used the appropriate denominator based on the number of observations available for the variable of interest (available-case approach), explaining minor differences in sample size across variables in Table 1.

Specifically, missing observations **(out of N = 3,614)** were as follows: **sex (n = 4; available n = 3,610), age (n = 2; available n = 3,612), residence (n = 107; available n = 3,507), TB clinical form (n = 6; available n = 3,608), TB status (n = 3; available n = 3,611), and treatment outcome (n = 109; available n = 3,505)**. Extrapulmonary TB localization was applicable only to extrapulmonary cases and was analyzed using its corresponding denominator **(n = 1,593).**

**Geocoding, spatial units and incidence estimation:**

Residential addresses were geocoded and linked to administrative polygons using ArcGIS (version 10.8, ESRI, Redlands, CA, USA). Spatial analyses were conducted at two levels: (i) province-wide analyses at the commune level (22 communes: two urban municipalities and twenty rural communes) and (ii) within Tétouan Municipality at the district level (18 urban districts).

Official administrative shapefiles were obtained from the Moroccan High Planning Commission (HCP; 2019 estimates) (**Supporting Information S4)**, and population denominators were sourced from the regional public health administration.

Annual TB notification rates were calculated for each spatial unit as the number of notified cases divided by the population at risk and expressed per 100,000 inhabitants. Average annual incidence rates for the 2019–2023 period were calculated by dividing the total number of cases by five years and the corresponding population denominator and expressed per 100,000 inhabitants.

Records with incomplete address information were manually reviewed. When at least the commune of residence could be identified, cases were assigned accordingly (and to the corresponding district for urban cases when district-level information was available). Cases with insufficient address information to allow assignment at least at the commune level (n = 107)—including homeless individuals and patients diagnosed in prisons—were excluded from spatial analyses but retained in descriptive analyses. All data were anonymized prior to analysis, and personal identifiers were removed to ensure confidentiality. The final anonymized dataset is provided as **Supporting Information (S2 Dataset)**.

**Time Series Decomposition:**

Monthly TB incidence time series **(2019–2023)** were decomposed into seasonal, trend, and residual components using **seasonal-trend decomposition based on LOESS (STL)** implemented in **R version 4.1.1 [13]. The LOESS approach allows modeling of nonlinear relationships and facilitates decomposition of time series into interpretable components**.

**Spatial Autocorrelation Analysis:** Spatial autocorrelation analysis was conducted to assess the degree of spatial dependence in TB incidence across geographic units. The stronger the influence of nearby observation values on each other, the higher the level of spatial correlation.

- Global spatial autocorrelation was used to explore the overall distribution of TB incidence in the studied area, reflecting the average degree of geographical aggregation of similar TB reported attributes using the global Moran’s I index, which is calculated using the Equation (1). When Moran's I is positive, it suggests that similar values tend to cluster together in space; when Moran's I is negative, indicates that spatial units with similar values are dispersed across space. When Moran's I = 0, the regions are randomly distributed and have no spatial correlation. Its statistical significance was tested according to the standardized statistic Z-score.

$$\begin{matrix} & \left( \mathbf{1} \right) I=\frac{n}{w}\times\frac{\sum_{i=1}^{n} *\sum_{j=1}^{n} *w_{\mathrm{ij}}\left( x_{i}-\overline{x} \right)\left( x_{j}-\overline{x} \right)}{\sum_{i=1}^{n} *\left( x_{i}-\overline{x} \right)^{2}} \\ & With w=\sum_{i=1}^{n} *\sum_{j=1}^{n} *w_{\mathrm{ij}} \end{matrix}$$

**Where** *I* is the Global Moran’s I statistic; ***n*** is the number of spatial units; ***x_i_*** and ***x*_j_** ​are the values of the studied variable (average annual tuberculosis incidence rate) in spatial units ***i*** and ***j***, respectively; ***x̄*** is the mean value of the studied variable across all spatial units; ***w_ij_*** ​ is the spatial weight between spatial units ***i*** and ***j****,* defined using a first-order Queen contiguity matrix; and ***w*** is the sum of all spatial weights.

- Local spatial autocorrelation was applied to identify spatial clustering within the study area. Hotspot analyses were conducted using crude average annual incidence rates and the Getis–Ord Gi* statistic (Equation 2) to detect hot spots (high-incidence areas) and cold spots (low-incidence areas). Local results were interpreted and visualized using standard confidence levels (90%, 95%, and 99%). Additionally, the Anselin Local Moran’s I index was used to provide a value for each spatial unit, and these values can be interpreted to identify different types of spatial patterns. These patterns are categorized on four types of clusters, namely, ‘high–high’ clusters (high-incidence areas surrounded by high-incidence areas); ‘low–low’ clusters (low-incidence areas surrounded by low-incidence areas); ‘high-low’ outlier (high incidence areas neighboring by low-incidence areas); and ‘low– high’ outlier (low-incidence areas neighboring by high-incidence areas).

Spatial relationships were defined using a first-order polygon contiguity weights matrix based on Queen contiguity (polygons sharing an edge or a corner) with row-standardized weights, as implemented in ArcGIS.

The Getis–Ord Gi* statistic was calculated as follows :

$$\begin{aligned} {\left( \mathrm{Equation} \mathbf{2} \right) G}_{i}^{*}=\frac{\sum_{j=1}^{n} *w_{\mathrm{ij}}x_{j}-\bar{x}\sum_{j=1}^{n} *w_{\mathrm{ij}}}{S\sqrt{\frac{n\sum_{j=1}^{n} *w_{\mathrm{ij}}^{2}-\left( \sum_{j=1}^{n} *w_{\mathrm{ij}} \right)^{2}}{n-1}}} \\ With S=\sqrt{\frac{\sum_{j=1}^{n} *x_{j}^{2}}{n}-{(\overline{x})}^{2}} \end{aligned}$$

**Where** G^∗​^_i_ is the Getis–Ord local statistic for spatial unit ***i***; ***n*** is the total number of spatial units; *x****_j​_*** is the value of the studied variable (average annual tuberculosis incidence rate) in spatial unit ***j***; ***x̄*** is the mean value of the studied variable across all spatial units; *w_ij_*​ is the spatial weight between spatial units ***i*** and ***j***, defined using a first-order Queen contiguity matrix; and ***S*** is the standard deviation of the studied variable.

***All spatial statistics (Global Moran’s I, Anselin Local Moran’s I, and Getis–Ord Gi*) were computed using ArcGIS Pro (version 10.8, ESRI, Redlands, CA, USA), with statistical significance assessed using 999 random permutations.***

**Spatio-temporal Scan Analysis:** Spatio-temporal clustering of TB incidence in Tétouan Province (2019–2023) was assessed using Kulldorff’s retrospective space-time scan statistic implemented in SaTScan (version 10.2.5). This method identifies clusters by scanning a cylindrical window, where the base represents the spatial extent and the height represents the temporal duration.

Analyses assumed a discrete Poisson model appropriate for count data with known population denominators. Monthly aggregation was used (time aggregation unit = 1 month), and analyses were conducted at both the commune and district levels. The maximum spatial cluster size was set to 30% of the population at risk, and the maximum temporal cluster size to 50% of the study period. Statistical significance was assessed using 999 Monte Carlo replications.

Clusters with the largest log-likelihood ratio (LLR) and p < 0.05 were classified as most likely clusters, while other statistically significant clusters were classified as secondary clusters. Relative risk (RR) was calculated as the ratio of risk inside versus outside each scanning window (equation **3**). To avoid spatial overlap, secondary clusters were identified using the SaTScan “No Geographical Overlap” option.

$$\left( Equation 3 \right) RR=\frac{observed cases in the cluster / expected cases in the cluster}{observed cases outside the cluster / expected cases outside the cluster}$$
