# Supplementary material for: Spatiotemporal patterns of tuberculosis in urban slums and urban–rural transition zones: evidence from Tétouan, Morocco, 2019–2023
Source: PLOS Glob Public Health. 2026 Apr 20;6(4):e0006315. doi: 10.1371/journal.pgph.0006315 (PMC13095008; doi:10.1371/journal.pgph.0006315)
Supplement: Attachment — Submitted filename: Suggestions on TB spatiotemporal analaysis.pdf [file pgph.0006315.s008.pdf]

Spatiotemporal patterns of tuberculosis within the urban slums and expanding urban–rural transition zones: lessons from Tétouan, Morocco (2019–2023)

Thank you for the opportunity to review a paper from Journal of Health, Population and Nutrition

Tuberculosis remains a threat in the Africa continent with little knowledge on its spatiotemporal patterns; therefore, it is an interesting area to look at. However, I appreciate the authors for their effort to discover the happening around this problem in Morocco. I also thank them for the opportunity to review their manuscript, as I enjoyed reading it. Besides, I have some comments to make, which are below.

Comments

Title:

This should be refine to Spatiotemporal Patterns of Tuberculosis in Urban Slums and Urban–Rural Transition Zones: Evidence from Tétouan, Morocco (2019—2023)

1. Abstract

   The results reported are unclear. For instance, “the average incidence was 103 per 100,000”. The question here is average incidence of what?

2. Method

   The authors stated in lines 131–132 that additional analysis includes “Regression (binomial and multinomial), comparison (Student’s t-test), and correlation (Pearson’s r) tests were conducted using SPSS Statistics v26.” What was the outcome variable? How did the authors define the outcome variable in the multinomial analysis? In all, none of these particularly multinomial analyses were captured throughout the manuscript.

3. Results

   Although authors should stick to reporting percentages for descriptive statistics, the findings reported do not correspond with the figures provided in Table 1. For instance, the authors stated in line 147 that “the median age was 40”, but in Table 1 of Line 389, it was rather the mean age. Also, in Line 147 the ratio of male-to-female is not “1.69” as reported by the authors but 1.70 (male=2272 and female=1338). Besides, in lines 150–151, “Five patients (0.1%) were homeless, and 42 (1.2%) were diagnosed in correctional facilities.” This is not corresponding with evidence provided in Table 1. In Line 389 of Table 1, the number of homeless is 4, and instead of patients diagnosed in prison, the authors reported correctional facilities. Percentages in Table 1 are not also adding up to 100%, even though some are reasonable. The column names of Table 1 are not understandable, with the figures provided for age groups being confusing. Notwithstanding, the sample size (n) of EPTB does not match with that of EPTB Localisations in Table 1.

In line 392, figures reported in Table 2 are also unclear, as a comma is used instead of probably a dot to indicate decimals. Also, analysing the Moran's I of a pooled 2019—2023 is redundant.

4. General
   The analytical approaches stated in the method without a given output are questionable. Again, the inconsistency of findings in the manuscript is regarded as a major issue and warrant the rejection this manuscript in this journal. Authors may consider reanalysing the data for consideration in other journals.
