# Supplementary material for: Spatiotemporal patterns of tuberculosis in urban slums and urban–rural transition zones: evidence from Tétouan, Morocco, 2019–2023
Source: PLOS Glob Public Health. 2026 Apr 20;6(4):e0006315. doi: 10.1371/journal.pgph.0006315 (PMC13095008; doi:10.1371/journal.pgph.0006315)
Supplement: Attachment — Submitted filename: Responses to editor and reviewers.pdf [file pgph.0006315.s011.pdf]

**Corresponding author: Ayoub Ez-Zari**
Abdelmalek Essaâdi University,
Faculty of Science of Tetouan, Department of Biology,
Laboratory of Biology and Health, Food Science and Health
Research Team (UAE/U16FS),
BP 2121, 93002, Tetouan, Morocco
ayoub.ezz1990@gmail.com
07-01-2026

**To: Editor-in-Chief** (PLOS Global Public Health)

**Subject: Response to reviewers and revised manuscript – Spatiotemporal patterns of tuberculosis within the urban slums and expanding urban–rural transition zones: lessons from Tétouan, Morocco (2019–2023)"** (Manuscript ID: PGPH-D-25-02866).

**Dear Editor-in-Chief,**

We are pleased to submit the revised version of our manuscript entitled: **"Spatiotemporal patterns of tuberculosis within the urban slums and expanding urban–rural transition zones: lessons from Tétouan, Morocco (2019–2023)"** (Manuscript ID: PGPH-D-25-02866), for consideration in *PLOS Global Public Health*.

We sincerely thank you and the reviewers for your thoughtful and constructive comments, which have helped us improve the quality and clarity of our work.

We have carefully addressed all reviewer and editor comments. A detailed, point-by-point response to each comment is provided below, and changes in the manuscript have been tracked for clarity.

We hope that the revisions meet your expectations and look forward to your favorable consideration.

# Comments and responses:

EDITOR:

Thank you for your submission. I have suggested major revisions because TB in Morocco is such an under-reported on topic. However, I note that both the reviewers (privately to me and in their comments to you) have raised significant concerns about your analytic process. In order to proceed toward publication, it is imperative that these are addressed thoroughly. This may include consulting additional experts on spatiotemporal mapping of TB disease for their guidance.

## Response:

Dear Dr. Hoddinott,

Thank you for your thoughtful handling of our manuscript and for the opportunity to resubmit our revision. We appreciate your comments and guidance, and we agree that tuberculosis in Morocco—and particularly in the northern region, including Tétouan—remains under-represented in the published literature.

We have carefully reviewed all reviewers' comments and your guidance, with particular attention to concerns regarding the analytic process. In the revised submission, we have:

1. Re-checked all data management steps, including inclusion/exclusion criteria, handling of missing data, and variable definitions.
2. **Re-run the full statistical and spatiotemporal analyses**, now in collaboration with the second author, a TB mapping specialist.
3. Re-generated all tables and figures directly from the final cleaned dataset to ensure complete internal consistency.

Specifically regarding spatial analysis, we:

- Re ran all spatial statistics using **SaTScan version 10.2.5** (previously 10.1.2), with results largely consistent with the previous analysis. Retracted one secondary cluster (Dersa III), as its *p*-value was above the 0.05 threshold. Detailed results of SaTScan runs are provided in the updated **Supporting Information S4 (Zip file)**.
- Reshaped **Figure 1** into two distinct panels (A and B) for greater clarity and interpretability.

These revisions include clarifications of the analytical pipeline and corrections of reporting inconsistencies. These revisions strengthen transparency, reproducibility, and interpretability, while preserving the study's main conclusions.

To further ensure methodological rigor, we consulted additional expertise in TB epidemiology through an academic TB research unit established by our team. This group includes clinicians (pulmonology), epidemiologists, and statisticians from Abdelmalek Essaâdi University and international partners, working in coordination with the region's TB reference center. We believe this strengthened input improved the clarity and defensibility of our analytic approach and reporting.

A detailed, point-by-point "Response to Reviewers" document is provided, including section references to all changes in the revised manuscript, in line with recommended peer-review response practice.

Reviewer #1:

This is an important area of research in TB and the data from the national programme has been well utilized. Though the study does not add much to the existing knowledge, it documents the process of shifting epidemiology. Some additional details however would make the interpretation of the results better.

**Response:** We thank the reviewer for the constructive assessment and for noting the relevance of using routine National TB Program data. This work is among the first to apply combined spatiotemporal methods to routine TB surveillance data in Morocco, while providing the first district/commune-level assessment for Tétouan Province to our knowledge. In response to the reviewer's suggestion that additional details would improve interpretation, we revised the manuscript to add key contextual and methodological information and to clarify how the findings extend existing knowledge at a fine geographic scale.

Specifically, in the revised manuscript we:

- Clarified the novelty and added-value as a fine-scale (district/commune) multi-year spatiotemporal analysis in Tétouan Province, including separate analyses for all TB and PTB to better reflect transmission dynamics and the potential need for forecasting (**Introduction/Discussion**).

- Expanded programmatic context (e.g., diagnostic approach and the potential influence of COVID-19 disruptions on notifications) to support interpretation of temporal trends (**Discussion**).
- Clarified inclusion/exclusion criteria and reporting consistency (e.g., totals by variable, missingness, and corrected descriptive-statistics reporting) and regenerated tables/figures from the cleaned analytic dataset (**Methods/Results; Tables**).
- Added/expanded methodological details for the spatiotemporal analyses (Moran's I / Getis-Ord Gi* / SaTScan settings) and pointed readers to Supporting Information containing full parameters and definitions (**Methods; Supporting Information**).

We hope these changes address the reviewer's request for additional detail and improve the clarity and strength of the interpretation.

1. Whether active case finding was performed in the years for which the data has been used? If yes, what were the approaches, strategies and methods? The number of cases diagnosed and the areas they belonged to would depend on the populations and groups screened.

**Response:** We thank the reviewer for this important point. During 2019–2023, no additional province-specific active case-finding activities were implemented beyond the routine TB control activities conducted under the Moroccan National Tuberculosis Control Program.

All TB cases notified in Tétouan Province are registered through the provincial Diagnostic Center for Tuberculosis and Respiratory Diseases (CDTMR), which serves as the reference center within the national TB control network; patients diagnosed outside the CDTMR (including in the private sector) are referred to the CDTMR for registration and standardized management. Therefore, the dataset analyzed represents routine programmatic TB notifications for the entire province, rather than cases generated through targeted screening of selected populations or neighborhoods.

2. Were there other strategies introduced into the programme during the study period, which might have an impact on the incidence of TB? Many countries had a change from alternate to daily regimen, which led to reduction in recurrent TB.

**Response:** We thank the reviewer for this important point and agree that changes in TB program strategies (including treatment regimen policies) can influence TB notification patterns and recurrence over time. During 2019–2023, no additional province-specific strategies were introduced in Tétouan beyond the routine policies and procedures implemented under the Moroccan National Tuberculosis Control Program (confirmed through the CDTMR, where the first author is Head of the Diagnostic Laboratory).

In Tétouan Province, the Diagnostic Center for Tuberculosis and Respiratory Diseases (CDTMR) functions as the provincial reference center within the national TB control network: once TB is confirmed, the patient is registered at the CDTMR and provided with a treatment card, after which treatment is delivered and adherence is monitored through the nearest primary health facility in line with the DOTS-based National TB Program (Directly Observed Treatment, Short-course", the WHO-recommended TB control strategy that combines standardized treatment with a structured system for follow-up and recording/reporting).

Medications are typically dispensed on a weekly basis in our setting, and patients are referred back to the CDTMR in case of suspected treatment interruption, major adverse events, or other clinical concerns; this organization of care was maintained throughout the entire study period.

3. What proportion of the TB patients were retreatment cases?

**Response:** We thank the reviewer for this comment. Treatment history (TB status) was available for 3,611 patients in our registry. Among these, retreatment TB cases (previously treated) represented 7.1% (259/3,611) of notified cases during 2019–2023. Retreatment cases were further classified as follows: 5/259 (1.93%) relapse after treatment failure, 77/259 (29.73%) retreatment after loss to follow-up and 177/259 (68.34%) retreatment after previous treatment completion.

To address this point more explicitly, we added these retreatment categories and their proportions to **Table 1** (treatment history/TB status). In addition, their definitions in **supplementary methods** as well.

4. How many patients had missing information and therefore were excluded from the analysis?

**Response:** We thank the reviewer for this comment. No notified TB patients were excluded from the study due to missing information; the full dataset included 3,614 cases notified during 2019–2023. Because missing data were limited and variable-specific, we used an available-case approach (i.e., each analysis used the appropriate denominator based on the number of observations available for that variable), which explains the slight differences in *n* across variables in Table 1. Specifically, the number of missing observations (out of N=3,614) was: gender 4 (n=3,610), age 2 (n=3,612), habitat 107 (n=3,507), TB type 6 (n=3,608), TB status 3 (n=3,611), and treatment outcome 109 (n=3,505). EPTB localization was only applicable to EPTB cases and was available for 1,593 patients; therefore, it was analyzed and presented with its corresponding denominator.

We clarified this missing-data handling strategy in the **Methods section** and added a short note in **Table 1 (and/or its footnote**) indicating that denominators vary because analyses were performed using available data for each variable.

Reviewer #2:

Tuberculosis remains a threat in the Africa continent with little knowledge on its spatiotemporal patterns; therefore, it is an interesting area to look at. However, I appreciate the authors for their effort to discover the happening around this problem in Morocco. I also thank them for the opportunity to review their manuscript, as I enjoyed reading it. Besides, I have some comments to make, which are below.

Comments

Title:

This should be refine to Spatiotemporal Patterns of Tuberculosis in Urban Slums and Urban–Rural Transition Zones: Evidence from Tétouan, Morocco (2019—2023)

**Response:** We thank the reviewer for this helpful suggestion. We agree that the proposed title is clearer and better reflects the focus of our study and the nature of our findings (i.e., presenting evidence rather than “lessons learned”) regarding urban slums and urban–rural transition areas, as well as the study period. Accordingly, we have revised the manuscript title to: “Spatiotemporal Patterns of Tuberculosis in Urban Slums and Urban–Rural Transition Zones: Evidence from Tétouan, Morocco (2019–2023)”

1. Abstract

The results reported are unclear. For instance, "the average incidence was 103 per 100,000". The question here is average incidence of what?

**<u>Response</u>:** We thank the reviewer for highlighting this lack of clarity. For clarity, we now report the average annual incidence (new cases) rate of all TB forms in Tétouan Province over the study period (2019–2023) = 113 per 100,000 population. We have revised the Abstract Results to explicitly state this and avoid ambiguity.

2. Method:

The authors stated in lines 131–132 that additional analysis includes "Regression (binomial and multinomial), comparison (Student's t-test), and correlation (Pearson's r) tests were conducted using SPSS Statistics v26." What was the outcome variable? How did the authors define the outcome variable in the multinomial analysis? In all, none of these particularly multinomial analyses were captured throughout the manuscript.

**<u>Response</u>:** We thank the reviewer for this careful reading and fully agree that, this statement is not consistent with the analyses reported in the manuscript. The paragraph referring to "Additional analyses: Regression (binomial and multinomial), comparison (Student's t-test), and correlation (Pearson's r) tests…" was inadvertently retained from a broader TB research project conducted by our research unit (including analyses on determinants of loss to follow-up) and is not part of the present spatiotemporal study.

Therefore, no outcome variable was defined for binomial or multinomial regression in this manuscript, and these analyses were neither performed nor intended to be presented here. We have removed this paragraph from the Methods section and ensured that the statistical methods now describe only the temporal, spatial, and spatiotemporal analyses actually conducted in this study (with full details provided in Methods **Supporting Information S3**)

3. Results

Although authors should stick to reporting percentages for descriptive statistics, the findings reported do not correspond with the figures provided in Table 1. For instance, the authors stated in line 147 that "the median age was 40", but in Table 1 of Line 389, it was rather the mean

age. Also, in Line 147 the ratio of male-to-female is not "1.69" as reported by the authors but 1.70 (male=2272 and female=1338). Besides, in lines 150–151, "Five patients (0.1%) were homeless, and 42 (1.2%) were diagnosed in correctional facilities." This is not corresponding with evidence provided in Table 1. In Line 389 of Table 1, the number of homeless is 4, and instead of patients diagnosed in prison, the authors reported correctional facilities. Percentages in Table 1 are not also adding up to 100%, even though some are reasonable. The column names of Table 1 are not understandable, with the figures provided for age groups being confusing. Notwithstanding, the sample size (n) of EPTB does not match with that of EPTB Localizations in Table 1.

**Response:** We thank the reviewer for this careful reading and agree that Table 1 and the corresponding Results text required clarification, harmonization, and improved presentation. We apologize for these inconsistencies; they arose during manuscript preparation when Table 1 was reformatted and updated using variable-specific denominators, while parts of the Results text were drafted from an earlier version of the descriptive outputs and were not fully synchronized with the final table. We have revised both the Results section and Table 1 to ensure full consistency, clearer labeling, and standardized rounding/terminology throughout, and we re-ran and cross-checked all descriptive analyses to confirm that all reported values now match the revised Table 1 exactly.

Specifically, we implemented the following corrections: (i) the Results previously referred to the "median age" while reporting a standard deviation (±19.1) and Table 1 reported the mean age (SD); we corrected the Results to report the mean age (SD) consistently with Table 1, (ii) we recalculated and corrected the male-to-female ratio using the counts in Table 1, (iii) we corrected the number/percentage of homeless individuals in the Results to match Table 1 and standardized terminology for individuals diagnosed in prisons/correctional facilities, and (iv) we reformatted Table 1 (column headers, parentheses, and age-group presentation) and explicitly stated denominators for each variable to avoid confusion when percentages do not sum to exactly 100% because of rounding and/or missing data.

Regarding the apparent discrepancy between the sample size (n) for EPTB and the denominator used for "EPTB localizations," we acknowledge that the original presentation was confusing and warranted clarification. In our routine surveillance database, cases with both pulmonary and extrapulmonary involvement are classified as PTB in the primary "TB type" field

(reflecting programmatic classification when pulmonary disease is present), while their extrapulmonary site(s) are still recorded in the "EPTB localization" field. This rule explains why the denominator for EPTB localizations may exceed the number classified as EPTB in the primary TB type variable in the original Table 1. To address this, we have revised Table 1 to clarify the classification and subdivided the TB clinical form into three subsections: PTB, EPTB, and recurrent EPTB/PTB. We have also ensured that the Results text aligns exactly with the corrected table.

In line 392, figures reported in Table 2 are also unclear, as a comma is used instead of probably a dot to indicate decimals. Also, analysing the Moran's I of a pooled 2019—2023 is redundant.

**Response:** We thank the reviewer for this comment. We agree that Table 2 was unclear because commas were used as decimal separators; this reflected our local/French software settings during data export, and we have now replaced commas with dots for all decimal values (Moran's I, Z-scores, and p-values) to comply with the journal's formatting conventions.

Regarding the Global Moran's I reported for "2019–2023," we respectfully disagree that it is redundant. The year-specific Moran's I values describe annual variation in spatial autocorrelation, whereas the pooled 2019–2023 statistic was included as a summary measure of the overall spatial dependence across the entire study period, computed from a single attribute per spatial unit (commune/district) representing the multi-year average annual TB notification rate. This multi-year rate was calculated as the total number of notified TB cases during 2019–2023 divided by the sum of the annual population denominators for 2019–2023 (i.e., an approximation of the person-time at risk while accounting for year-to-year population changes). We have clarified this definition by revising the table caption/row label and adding a **footnote to Table 2** to ensure an unambiguous interpretation.

4. General

The analytical approaches stated in the method without a given output are questionable. Again, the inconsistency of findings in the manuscript is regarded as a major issue and warrants reanalysing the data for consideration in other journals.

**Response:** We thank the reviewer for this important general comment. We agree that all analytical approaches described in the Methods must correspond to clearly reported outputs. In

the revised manuscript, we re-ran the full analytical workflow and verified complete consistency between datasets, statistical outputs, tables, figures, and Results text.

The previously mentioned regression and correlation analyses were inadvertently retained from a separate project and were neither performed nor required for this spatiotemporal study. This text has now been removed to ensure full alignment between Methods and Results.

The remaining analyses (temporal decomposition, Global and Local Moran's I, Getis–Ord Gi*, and SaTScan) have been fully rechecked and are consistently reported in the revised manuscript and Supporting Information.
