# Supplementary material for: Spatiotemporal patterns of tuberculosis in urban slums and urban–rural transition zones: evidence from Tétouan, Morocco, 2019–2023
Source: PLOS Glob Public Health. 2026 Apr 20;6(4):e0006315. doi: 10.1371/journal.pgph.0006315 (PMC13095008; doi:10.1371/journal.pgph.0006315)
Supplement: Attachment — Submitted filename: PLOS_pgph_d_25_02866_review_TB.pdf [file pgph.0006315.s010.pdf]

# Review of Spatiotemporal Patterns of Tuberculosis in Urban Slums and Urban–Rural Transition Zones: Evidence from Tétouan, Morocco (2019–2023)

The authors present a spatiotemporal analysis of notified Tuberculosis (TB) cases in areas of Morocco for the period 2019-2023, differentiating between pulmonary TB (PTB) and extrapulmonary TB (EPTB). The study assessed seasonal and temporal trends, spatial dependence of cases, and space–time clustering in SaTScan.

The paper represents an almost complete revision of the original paper addressing multiple comments and suggestions.

- The title was revised.
- The abstract has been revised and restructured, removing subheadings.
- The statistical and spatiotemporal analyses were re-run, in collaboration with a TB mapping specialist.
- All figures and tables were regenerated from the new dataset.
- The analysis now combines routine TB surveillance data in Morocco, in a novel, fine-scale, multi-year, spatiotemporal analysis, including separate analyses for both PTB and EPTB, reflecting transmission dynamics.
- Retreatment categories and proportions were added in Table 1 as requested by previous reviewers.
- Tables and text now correspond.
- I support the decision to retain the year-specific Moran's I values to describe annual variation in spatial autocorrelation.

Some minor comments and suggestions:

- Both terms: spatiotemporal and spatio-temporal are used, maybe standardize?
- Figures 3&4 – in all maps the text is too small to read. It would maybe be more helpful to assign a letter for labelling to each area, and cross reference to the relevant letter when mentioning this area in text.

I would support publication of this article.
