# Supplementary material for: Spatiotemporal patterns of tuberculosis in urban slums and urban–rural transition zones: evidence from Tétouan, Morocco, 2019–2023
Source: PLOS Glob Public Health. 2026 Apr 20;6(4):e0006315. doi: 10.1371/journal.pgph.0006315 (PMC13095008; doi:10.1371/journal.pgph.0006315)
Supplement: Attachment — Submitted filename: Responses_to_editor_and_Reviewers_auresp_2.pdf [file pgph.0006315.s012.pdf]

**Corresponding author: Ayoub Ez-Zari**
Abdelmalek Essaâdi University,
Faculty of Science of Tetouan, Department of Biology,
Laboratory of Biology and Health, Food Science and Health
Research Team (UAE/U16FS),
BP 2121, 93002, Tetouan, Morocco
ayoub.ezz1990@gmail.com
07-01-2026

**To: Editor-in-Chief** (PLOS Global Public Health)

**Subject: Response to reviewers and revised manuscript – Spatiotemporal Patterns of Tuberculosis in Urban Slums and Urban–Rural Transition Zones: Evidence from Tétouan, Morocco (2019–2023)"** (Manuscript ID: PGPH-D-25-02866R1).

**Dear Editor-in-Chief,**

We are pleased to submit the revised version of our manuscript entitled, "Spatiotemporal patterns of tuberculosis within the urban slums and expanding urban–rural transition zones: Evidence from Tétouan, Morocco (2019–2023)" (Manuscript ID: PGPH-D-25-02866R1), for consideration in PLOS Global Public Health.

We sincerely thank you and the reviewers for your thoughtful and constructive comments, which have greatly helped us improve both the quality and clarity of our work.

We have carefully addressed all reviewer and editor comments. A detailed, point-by-point response to each comment is provided below, and all changes in the manuscript have been tracked for transparency.

Additionally, we have thoroughly reviewed the reference list to ensure it is complete and accurate. Any retracted articles have been removed or replaced with relevant, current references. For any retained retracted articles, we have indicated their retracted status in the References list and included the full citation of the retraction notice, as recommended.

Furthermore, Figures 3 and 4 have been revised to improve readability, and the supplementary materials have been reorganized and updated to ensure full compliance with the journal's formatting and submission guidelines.

We hope that the revisions meet your expectations and look forward to your favorable consideration.

**<u>Comments and responses</u>:**

**<u>Review #3</u>**

Reviewer #3: This is a nice paper describing the spatial-temporal epidemiology of TB in specific part of Morocco. It appears that the authors have responded thoroughly to previous reviewer comments. I have a few suggestions and questions.

Abstract:

1. There are some relative risks provided in the abstract but they need to be accompanied by confidence intervals so readers understand the accuracy. Also, the first time an RR is presented it says "1.90-2.03" but it is unclear if that is a range and if so what type of range. The third instance, it says "RR up to 3.00", this is unclear, a single RR should be provided with a confidence interval.

**<u>Response</u>:** We thank the reviewer for this comment. SaTScan is primarily a cluster detection and hypothesis-testing tool rather than a classical parameter-estimation framework. Its outputs focus on the identification of statistically significant clusters and include the relative risk (RR) for each detected cluster, the log likelihood ratio (LLR), and p-values derived from Monte Carlo simulations.

Statistical inference in SaTScan is therefore based on hypothesis testing using Monte Carlo permutation tests, not on confidence interval estimation. Consequently, relative risks are provided as point estimates only, and 95% confidence intervals are not calculated or reported. Moreover, the RR estimated by SaTScan is conditional on the detected cluster, which itself results from a scanning and maximization procedure; under these conditions, classical confidence intervals would not be statistically valid without explicitly accounting for the cluster selection process.

In light of this, the spatial scan statistic implemented in SaTScan does not estimate 95% confidence intervals for relative risks. To avoid any ambiguity, we revised the Abstract to explicitly indicate the inferential approach used by adding the wording *"using Monte Carlo permutation tests implemented in SaTScan"* and by reporting relative risks rang explicitly associated with their p-values (*"relative risks (RR) ranging from 1.90 to 2.03; p < 0.01"*).

These changes clarify that uncertainty was assessed through hypothesis testing rather than confidence interval estimation.

Methods:

2. A little more clarity on the spatial units of analysis is needed. The description says there are two urban municipalities and 20 rural districts and the urban area is divided into 18 districts. Is the unit of spatial analysis district/commune, i.e. a total of 38 areas? If so, this should be stated clearly (or the correct statement given if this is incorrect). Also, how many districts are in the old medina (slum Iike) area? Lower down it says that data were extracted and aggregated – aggregated in what way? To the spatial unit of analysis? Finally, how was urban defined?

**Response:** Thank you for this helpful comment. We have revised the Methods section to more clarify the spatial units of analysis, and the data aggregation process.

Administrative divisions and urban–rural classification followed the official 2019 framework of the *Haut Commissariat au Plan* (HCP) as well we used their urban-rural definitions. At the commune level, Tétouan Province comprises 22 administrative units**:** two urban municipalities (Tétouan and Oued Laou) and 20 rural communes. Spatial analyses at this scale were therefore conducted on these 22 units.

At a finer scale, district-level analyses were performed exclusively within the urban municipality of Tétouan, which is subdivided into 18 districts. Among these, seven districts correspond to the Old Medina**,** characterized by high population density, overcrowded housing, and poor infrastructure, and are commonly classified as slum-like areas, while the remaining 11 districts correspond to the New (modern) Medina. The names and characteristics of all districts are provided in Supporting Information S1: Supplement 2.

Regarding data aggregation, individual TB notification records were aggregated by spatial unit of analysis (district or commune, depending on the analysis) and by time unit (month and year) prior to statistical analysis. These clarifications have been added to the manuscript.

3. In the spatio-temporal scan section it says “Relative risk (RR) was calculated for each cluster.” It is unclear what this relative risk is. What was the outcome and what is the risk relative to (i.e. what is the comparison group)?

We thank the reviewer for this comment. In the discrete Poisson model used in SaTScan, the relative risk (RR) for each cluster represents the risk within the cluster relative to the risk outside the cluster. Specifically, RR is calculated as:

$$RR = \frac{\text{observed cases in the cluster / expected cases in the cluster}}{\textit{observed cases outside the cluster / expected cases outside the cluster}}$$

So it compares the incidence within the cluster to the incidence outside the cluster. This is standard for Poisson-based scan statistics but needs to be explicitly stated for readers unfamiliar with SaTScan. This definition has now been added to the Methods section to clarify the outcome and the comparison group.

Table 2:

4. I recommend using fewer decimal places here? Maybe two would be best. Otherwise, it is hard to read this table with so many that are unnecessary.

**Response:** We thank the reviewer for this suggestion. The number of decimal places has been reduced to improve readability, in line with common reporting standards for spatial autocorrelation statistics.

Results:

5. Several RRs are presented but they need to be accompanied by confidence intervals.

**Response:** We thank the reviewer for this comment. The relative risks reported in this study were estimated using the spatial scan statistic implemented in SaTScan, which is designed for cluster detection and hypothesis testing rather than parameter estimation. As such, SaTScan provides relative risks as point estimates and does not compute 95% confidence intervals. Statistical inference is instead based on Monte Carlo permutation testing, with significance assessed using p-values.

Table 3:

6. Again, confidence intervals need to be presented. These should replace the LLRs, which are not very meaningful to readers.

**Response:** We thank the reviewer for this comment. In the spatial scan statistic implemented in SaTScan, the log likelihood ratio (LLR) is the fundamental test statistic used to identify, rank, and evaluate the statistical significance of clusters. Confidence intervals cannot replace LLRs, as they address a different inferential objective and are not estimated within this framework.

SaTScan is a cluster detection and hypothesis-testing method rather than a parameter-estimation approach; relative risks are therefore reported as point estimates, and statistical significance is assessed using Monte Carlo permutation testing rather than confidence intervals. LLRs are routinely reported in spatial scan articles and provide essential information on the strength and ranking of detected clusters. To improve clarity for readers, we ensured that all reported relative risks are accompanied by their corresponding p-values and that the use of Monte Carlo-based inference is clearly stated in the Abstract and Methods.

Discussion:

7. Sentence in lines 307-9 starting “Patterns of population movement…” should have references to support these suggested movement patterns.

**Response:** We thank the reviewer for this comment. We have added appropriate references to support the statement on population movement and tuberculosis transmission. Specifically, we now cite evidence from the World Health Organization Global Tuberculosis Report and peer-reviewed studies documenting the role of rural-to-urban migration, cross-border population movement, and urban slum environments in increasing TB exposure and transmission risk (Oppong et al., 2015; Dhavan et al., 2017; Noykhovich et al., 2019; WHO, 2024). These references have been added to lines 307–309 of the revised manuscript.

8. Although the authors mention the limitation of COVID-19 related under-reporting, they don’t mention general under-reporting which could affect their conclusions more broadly. By which I mean the fact that they are presenting notification data and therefore, low incidence might reflect under reporting rather than genuinely low incidence and similarly, high incidence might reflect more thorough case-finding and identification of cases, which would be a positive thing.

**Response:** We thank the reviewer for this important comment. In our setting, tuberculosis notification is centralized, as all patients with suspected TB are referred to the sole TB

diagnostic and treatment center in the city. In addition, patients with microbiologically confirmed TB diagnosed in private medical laboratories are systematically referred to this center, which is the only facility authorized to initiate treatment, maintain treatment records, and conduct patient follow-up, regardless of whether the diagnosis was made in the public or private sector.

Since 2017, GeneXpert has been used as the standard first-line diagnostic test for all presumptive TB cases. The transition from partial to full implementation of GeneXpert as a diagnosis tool was associated with a substantial increase in notifications, reflecting improved diagnostic sensitivity rather than changes in transmission. During the study period (2019–2023), all suspected cases were eligible for GeneXpert testing and chest radiography in our center, a combination that ensures high sensitivity for case detection.

Under these conditions, systematic under-reporting of TB cases is considered unlikely in our study area. We agree, however, that notification data may still be influenced by variations in healthcare access and case-finding intensity, and we have clarified this point in the revised manuscript. Apart from the documented disruptions related to the COVID-19 pandemic, we do not believe that general under-reporting substantially affected our conclusions.

Figure 1:

9. The legend uses the word "evolution" but that is not what is being shown in this figure, it is just a stratification of the temporal trends by clinical form.

**Response:** We thank the reviewer for this comment and agree that the term "evolution" was imprecise. The figure legend has been revised to refer to temporal trends stratified by clinical form and place of residence.

**Review #4**

The authors present a spatiotemporal analysis of notified Tuberculosis (TB) cases in areas of Morocco for the period 2019-2023, differentiating between pulmonary TB (PTB) and extrapulmonary TB (EPTB). The study assessed seasonal and temporal trends, spatial dependence of cases, and space–time clustering in SaTScan.

The paper represents an almost complete revision of the original paper addressing multiple comments and suggestions.

• The title was revised.

• The abstract has been revised and restructured, removing subheadings.

• The statistical and spatiotemporal analyses were re-run, in collaboration with a TB mapping specialist.

• All figures and tables were regenerated from the new dataset.

• The analysis now combines routine TB surveillance data in Morocco, in a novel, fine-scale, multi-year, spatiotemporal analysis, including separate analyses for both PTB and EPTB, reflecting transmission dynamics.

• Retreatment categories and proportions were added in Table 1 as requested by previous reviewers.

• Tables and text now correspond.

• I support the decision to retain the year-specific Moran's I values to describe annual variation in spatial autocorrelation.

**Some minor comments and suggestions:**

• Both terms: spatiotemporal and spatio-temporal are used, maybe standardize?

**Response:** Thank you for this comment. We have standardized the terminology throughout the manuscript and retained the "spatiotemporal" form consistently.

• Figures 3&4 – in all maps the text is too small to read. It would maybe be more helpful to assign a letter for labelling to each area, and cross reference to the relevant letter when mentioning this area in text.

**Response:** We thank the reviewer for this helpful suggestion. To improve readability and presentation, we have revised the spatial figures accordingly. Specifically, the original two figures have been reorganized and expanded into three separate figures, allowing for clearer visualization and improved layout of the maps within the manuscript.

We would also like to clarify that the commune names are now displayed in the maps and are readable, although they remain relatively small to preserve map clarity and avoid overcrowding. To further facilitate geographic interpretation, we have included a comprehensive descriptive map in Supplementary Figure S1, where all commune names are displayed at a larger and more legible scale. This supplementary figure is intended to assist readers in identifying locations and to enable easier cross-referencing with the areas discussed in the text. These changes have been implemented to ensure that the figures remain clear and informative while maintaining an appropriate balance between readability and visual clarity.

## **Licensing for Maps**

Dear, Editor-in-Chief Thank you for your message and for reviewing our submission.

In response to your request regarding the base map layers used in Figures 3–5, we confirm that the maps were generated using ArcGIS and that the administrative boundary base layers are derived from publicly available Morocco administrative boundary datasets distributed via the Humanitarian Data Exchange (United Nations Office for the Coordination of Humanitarian Affairs).

Source of the base layer:

https://data.humdata.org/dataset/cod-ab-mar

License information:

Creative Commons Attribution 4.0 (CC BY 4.0)

These datasets compile official administrative boundary information originating from Moroccan national statistical sources, including the Haut-Commissariat au Plan.

To ensure transparency, we have updated the figure legends for Figures 3–5 to include the source of the boundary data, the license information, and the software used to generate the maps.

Please let us know if any additional clarification is required.
